# Could the new dairy policy affect milk allocation to infants in Kenya? A best-worst scaling approach

**DOI:** 10.1016/j.foodpol.2021.102043

**Published:** 2021-05

**Authors:** Emmanuel Muunda, Nadhem Mtimet, Franziska Schneider, Francis Wanyoike, Paula Dominguez-Salas, Silvia Alonso

**Affiliations:** a International Livestock Research Institute, Box 30709, Nairobi, Kenya; b International Fund for Agricultural Development, 1191 - Cairo, Egypt; c University of Hohenheim; d Natural Resources Institute, University of Greenwich, Kent, UK; e International Livestock Research Institute, Box 5689, Addis Ababa, Ethiopia

**Keywords:** Kenya, Informal markets, Dairy, Regulations, Latent class model, Best-Worst Scaling

## Abstract

Milk is an important food item in the diet of Kenyans, especially infants. During the last two decades, the dairy sector in Kenya has witnessed important growth in production and improvements in milk quality. The informal marketing channel still prevails, and the Kenya Dairy Board, the regulator of the dairy sector, is currently introducing new regulations to increase registration and licensing of smallholder producers and dairy business operators, improve product hygiene and quality, and safeguard the health of consumers. These new regulations encompass, among others, the requirement to pasteurize milk before it is sold and adopt traceability processes and quality tests; most of these will probably result in higher milk prices at retail level. Using the best-worst scaling approach in this study, we analyzed the potential effects of milk price increase on household milk purchase and allocation to infants (6–48 months of age). The results indicate that an increase in milk price will decrease milk allocation to and intake by children. Households will replace the lost infant milk intake by fruits or porridge that might not be of equivalent nutritional value to milk. Any reforms to policies and regulatory systems aimed at streamlining the dairy sector should account for impacts on milk prices, responsiveness of consumers to price variations and infant nutrition. We recommend that regulatory and development agencies consider interventions that do not increase price for consumers and facilitate access to affordable and safe milk for children and entire households.

## Highlights

• The new dairy policies will reduce low-income households consumption of milk.
• The increase in milk price will decrease milk allocation and consumption by infants.
• Households revealed decisions making are close to the best-worst experiment results.
• Infants food substitutes to milk are generally of lower nutritional value.

## 1 Introduction

**Fig. 1:**
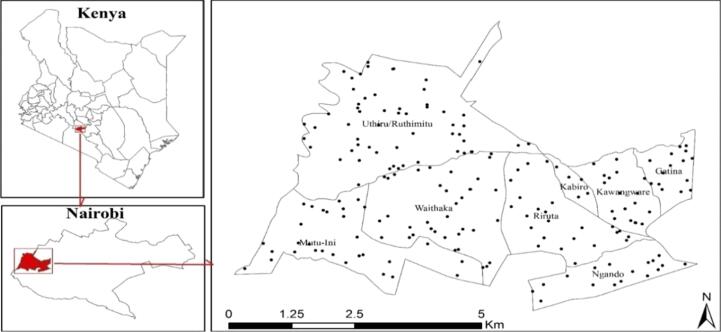
A map of the study area. The dots indicate the geo-spatial locations used for random sampling of households.

Kenya is ranked among the highest milk producing and consuming countries in Africa, with most of the milk produced by smallholder farmers. The country’s annual per capita consumption of milk has recently been estimated at 110 kg (Rademaker et al., 2016), with previous FAO study estimating consumption of 19 kg in rural areas and 125 kg in urban areas. This falls short of the widely recommended 220 kg annual per capita consumption (FAO, 2011, Odero-Waitituh, 2017) (Fig. 1.).

According to the Kenya Dairy Board (KDB), Kenyans consumed about four billion liters of milk in 2012 (MoALF, 2013). As per KDB’s records, the formal sector that markets industrially processed and packed milk recorded a total intake of about 495 million liters during the same year (KDB, 2017) representing 15% of the overall supply. These figures confirm the overall trend indicating that the majority of milk marketed in Kenya is sold through the so-called “informal dairy sector/channel” commercializing raw or otherwise non-industrially processed, unpacked milk (FAO, 2011). The high demand and preference for raw/non-industrially processed milk is mainly due to its wide availability and access, lower price, consumers’ perception of freshness, and better taste (Alonso et al., 2018a, Bebe et al., 2018, Blackmore et al., 2015, Bosire et al., 2017, Fadiga and Makokha, 2014, Njarui et al., 2011, Smallholder Dairy Project, 2004).

This higher demand and preference for informally marketed milk makes the informal dairy sector a critical player in meeting the consumption demand for milk in the country, especially among low-income households which rely on milk as an affordable source of animal protein (relative to other animal products), especially those with children below the age of five years. Several studies (Ayele and Peacock, 2018, Dominguez-Salas et al., 2016, Grace et al., 2018, Njarui et al., 2009) conducted among low-income households in Kenya with malnourished and/or stunted children have found that consumption of milk and/or other animal-source food improves the nutritional status of children.

Despite the dominance of the informal dairy sector in milk commercialization and the role it plays in household milk consumption, not only in Kenya but also in neighboring countries like Tanzania (Kilelu et al., 2017), Rwanda (IFAD, 2016) and Malawi (Revoredo-Giha & Renwick, 2016), regulatory agencies and other stakeholders in these countries have recently been promoting policy interventions geared towards eliminating it in favor of consolidating the formal dairy sector. Such policies are promoted on public health claims with an understanding that they will lead to improvements on milk quality and safety and greater compliance with international food safety standards, even though evidence on the public health effects of such policies is not available (Blackmore et al., 2020, Grace et al., 2014, Kang’ethe et al., 2020, Roesel and Grace, 2015).

In the Kenyan context, regulatory interventions criminalizing the sale of raw milk date back to 1958 when the Dairy Industry Act was enacted. Just like the case with the current policy efforts, this dairy Act aimed at addressing food safety and quality concerns (Leksmono et al., 2006). According to Leksmono et al. (2006), this regulation largely supported the large-scale producers and their urban markets. Until 2004 when there was a policy change (Kenya Subsidiary Legislation, 2004), informal dairy sector players were often harassed and operated without licenses despite their significant growth in numbers and market share (Leksmono et al., 2006, MoALF, 2013). However, the revised policy of 2004 allowed the KDB, following a training scheme, to train and license small-scale vendors as a pathway to formalization. This engagement entailed strengthening the capacity of informal traders in milk handling, value addition and business development. In a study conducted by Kaitibie et al. (2010) to assess the impact of this policy change, it was found that there was an increased number of licensed vendors and a high welfare benefit for actors across the dairy value chain with a net worth of USD230 million (Alonso et al., 2018a).

Despite these gains, since the training scheme, subsequent policy change efforts have shifted from attempting a gradual transformation of the informal sector through promoting better milk handling practices as envisioned in the policy change of 2004, to a more prescriptive approach based on regulatory requirements that are often not easily achievable in the informal dairy sector. Like the Dairy Act of 1958, recent policy reforms require industrial processing of milk before selling to consumers, effectively consolidating the formal sector and excluding the informal markets. It potentially leaves the informal dairy actors either as their suppliers at best or eliminated completely from the market at worst. Some argue this will streamline regulation and overall improve milk safety, although this has not yet been proven and existing evidence suggests industrially pasteurized milk is often no better at meeting standards than milk sold in the informal markets (Alonso et al., 2018a, Nyokabi et al., 2020, Omore et al., 2000, Alonso et al., 2018b). It will also potentially fail to address the welfare of over 80% of dairy sector actors holistically.

When policies that enhance food safety (a public good) and conform to international standards, are risk-based (i.e. reducing the risk to human health), feasible and affordable, they are not only beneficial to the dairy sector and market, but also valuable to consumers. Previous studies have actually shown positive impacts of transformative policy interventions on the microbiological quality of milk and general improvement of informal milk markets (Kaitibie et al., 2010, Leksmono et al., 2006). But the effect of such policies on health outcomes (i.e. reduced milk-borne disease) has never been studied. There is little available data on the health burden caused by consumption of unsafe milk in Kenya, but considering that most people in urban and *peri*-urban settings boil milk before consumption (Grace et al., 2008), and that boiling is known to be effective at inactivating microbial pathogens (e.g. Metwally et al., 2011), it is expected that consumption of milk from the informal markets may have a similar health burden to milk associated with formal markets. Nevertheless, these policies should also be designed with an inclusive and bottom-up approach including smallholder producers and informal milk traders/sellers and accompanied by government fund allocation and capacity building to uphold the transformation and formalization of the informal dairy sector.

Given the market share of the informal dairy sector in Kenya and its role in providing affordable milk of high nutritional value to low-income households, a ban on commercialization of raw milk would likely affect milk and nutrient intake and consequently, the health and nutrition outcomes for consumers, especially in the *peri*-urban areas where production is limited (Dominguez-Salas et al., 2016).

Policy or regulatory frameworks that either directly or indirectly have an impact on the prices and distribution of food items, food safety, or even affect consumer awareness regarding specific food products, influence the choice of consumers and consequently, the intake of the targeted product. As Ralston (1999) mentions, how policies and regulatory systems affect dietary choices depends on the effect of the policies on cost of production, the resulting real retail prices, responsiveness of consumers to price variations and the influence of the policy on consumer preference. The new regulations proposed by KDB currently under review stipulate that milk should be processed (preferably through industrial pasteurization process), chilled and transported using adequate transport means. It should also be traceable and subjected to milk safety and quality testing at different stages. These new regulations will lead to high processing and transaction costs, especially for the majority of informal small-scale milk traders and will likely result in substantial increase in milk prices.

While various researchers have studied the impact of policy change on production, market dynamics and consumers’ willingness to pay after diverse interventions (Blackmore et al., 2020, Kaitibie et al., 2010, Kumar et al., 2017), in our view, there is a knowledge gap on how such policy change can affect decisions of milk allocation to young children in low-income households and its impacts on their nutrition. The current study tries to fill this gap by assessing the potential impact of milk price increase, by analyzing, for example, how households’ milk allocation to infants between 6 and 48 months of age would be affected from a policy change banning raw milk marketing. In this study, we use the term “raw milk” to refer to milk from informal markets. This is done to make a clear distinction with the milk from the formal market which is pasteurized and was not included in our study. However, it is important to note that not all milk in Kenyan informal markets is sold raw; some of it may be boiled or even pasteurized before selling.

The findings of this study will be useful for policy and decision makers in Kenya and other countries in the region (Rwanda, Tanzania, Malawi, etc.) where the informal market is an important component of the dairy sector, and where policies are being developed to reduce its role in the market . The lessons learnt are also applicable to other animal-source food value chains (e.g. meat) that are facing similar issues related to food safety and informal markets (Roesel and Grace, 2015).

## 2 Methodology

### 2.1 Data collection

The study was carried out in Dagoretti area comprising Dagoretti North and South sub counties which lie to the West of Nairobi, Kenya. The study area is characterized by low-income informal settlements, some in *peri*-urban settings with limited agricultural activities and others in purely urban areas.

The survey was carried out in households randomly selected using geospatial random points generated by ArcGIS 10.4.1©. A protocol was used by field staff to guide the selection of eligible households closest to each selected random point. The study included 200 households that had at least one child within the age range of 6–48 months. The selection criteria for households also included purchase of milk from informal markets and have a monthly income not exceeding KES30,000 (USD300). For the purposes of this study, a household was defined as a group of people that take food from the same ‘(economic) basket’, in the same house for at least three months before the survey date.

The study was conducted between April and June 2017. A structured questionnaire was developed, pretested and revised to collect data on seven-day recall household purchase of milk and other dairy products, milk consumption by each household member, and perceptions on milk quality and safety. The survey also included questions on other food products consumed in the seven days preceding the interview day; household income and expenditure; and demographic characteristics like household size, composition, household members’ age and educational level.

### 2.2 Experimental design

To assess the likely effects of an increase in milk price on milk purchase and allocation of low-income households for their members including infants (6–48 months old) due to the new regulations, we used the best-worst scaling approach (Finn and Louviere, 1992, Marley and Louviere, 2005), which has over time gained attention in the agricultural and food demand fields (Auger et al., 2007, Bazzani et al., 2018, Campbell and Erdem, 2015, Caputo and Lusk, 2020, Costanigro et al., 2015, Erdem, 2018, Ochieng’ and Hobbs, 2016, Rao et al., 2019, Van Wezemael et al., 2014). The general assumption is that best and worst choices derive from a common underlying utility function, one that combines the best and worst choices into a single model that results in a separate score, or utility, for each item (Chrzan & Peitz, 2019). The statistical model underlying best-worst scaling assumes that the relative choice probability of a given pair is proportional to the distance between the two attribute levels on the latent utility scale (Flynn et al., 2007).

The experimental design for the best-worst scaling experiment is composed of nine choice cards presenting nine different intrahousehold milk allocation options that a household may practice (Table 1) including adjusting the amount of milk allocated, adjusting budgets and substituting milk with other food items. Where households were opting to substitute milk with other food items, they were asked to clarify the substitutes. The selection of the nine allocation options was based on literature review, previous studies conducted in the area and an initial scoping study that informed the various behavioral options.

**Table 1:** Options used in generating the choice cards.

No.	Option
O1	Decrease raw milk quantities for all family members without replacing it by any other food product
O2	Decrease raw milk quantities for all family members and replace it with another food product only for children < 4 years
O3	Decrease raw milk quantities for all family members and replace it with another food product for all family members EXCEPT for children < 4 years
O4	Decrease raw milk quantities for all family members and replace it with another food product for all family members
O5	Keep raw milk quantities the same for children < 4 years and decrease it for the rest of the family members
O6	Decrease the quantities of raw milk for children < 4 years without replacing it by other food products, and keep the same quantities of raw milk for adults
O7	Decrease the quantities of raw milk for children < 4 years, while replacing it by other food products, and keep the same quantities of raw milk for adults
O8	Keep buying the same quantities of raw milk by increasing milk budget
O9	Stop buying raw milk

**Fig. 2:**
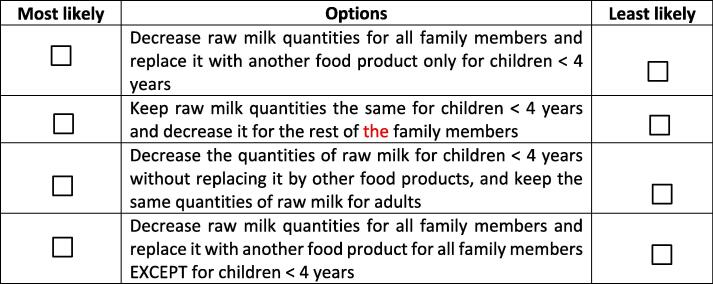
An example of a best-worst choice card.

The nine options were used to create nine different choice cards. Each best-worst choice card included four different alternatives (Fig. 2). We used the %MktBIBD Macro (Kuhfeld, 2010) of SAS 9.2 software to generate a nearly Balanced Incomplete Block Design - BIBD (Erdem et al., 2012, Hamada, 1973, Street and Street, 1996) of nine choice cards with a block design efficiency of 99.4%, an average pairwise frequency of 1.5. Each option appeared an equal number of times across the choice cards (four in this case). In a BIBD, each treatment (option in this study) appears the same number of times and each pair of treatments (options) appears the same number of times.

**Fig. 3:**
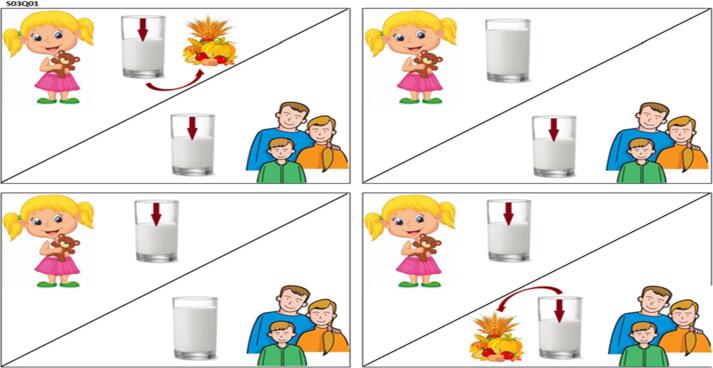
A pictorial presentation of the above best-worst choice card.

To facilitate the respondent task, we used choice cards in pictorial form (example in Fig. 3). The use of pictures was expected to increase the respondent’s ability to retain, understand and compare the offered alternatives. Pictorial cards were first explained to the respondents so that they could relate each picture with the milk allocation alternative that it described. For example, a glass of milk with an arrow pointing downwards indicated a decrease in milk consumption. When individual cards were presented to the participants as part of the interview, they were explained again. Pictures were used to represent the choices because they have been found to help participants understand the options more clearly than oral and written presentations of options (He, 2015).

For each choice card, the respondent was asked to select the most likely (best) option and the least likely (worst) option that she/he would choose in the event milk prices increase by 40% of the current price. Each respondent was presented with a total of nine best-worst choice cards. The choice of 40% increase in prices was based on the following: i. observed raw milk prices during the design of the survey (mean and median prices were respectively KES78/liter and KES76/liter); ii. various additional costs incurred by value chain actors because of the new regulations (additional cess payments and consumers safety levy, pasteurization costs, transport costs, costs of milk hygiene analysis and book records keeping, etc.); iii. the assumption that if the selling of raw milk to consumers is banned (that was discussed in the initial draft of the new regulations), then in the short run the new prices will be close to the ones of packaged milk (around KES100/liter).

**S03q01. “**If **raw milk** price increases by **40%** compared to high season prices, which corresponds to new raw milk price around **KES100/liter**, from the four options below please indicate which is the most likely option you will choose and the least likely option you will not choose? (Tick only one case as most likely and one case as least likely)”

### 2.3 Data analysis

#### 2.3.1 Best-worst scores

We have initially calculated standardized most-least scores (generally known as best-worst scores) to assess respondents’ stated importance of the various allocation alternatives and the importance of their respective levels. The standardized scores were calculated following Loose & Lockshin (2013) and Ochieng’ & Hobbs (2016). The details of the score calculations are reported in equation (a) in Appendix A. The obtained scores were transformed to a positive scale then standardized (equation (b), Appendix A). For further simplicity of interpretation, we standardized the square-root scale to add up to 100% as per equation (c) in Appendix A.

#### 2.3.2 Models estimation

Analyzing best-worst scaling data could also be based on probabilistic models depending on the process used by respondents in providing best and worst responses as described by Marley & Louviere (2005). Respondents could either first chose the best option and then the worst option (or vice versa) leading to a use of sequential model, or evaluate all possible pairs of options and simultaneously chose the pair of options that maximizes the difference between the best and the worst choices leading to the use of maxdiff model. In this study, we opted for the maxdiff model and we used both the mixed logit and the latent class models to estimate the data. More details about these models are reported in Appendix B. For the latent class model, to determine the appropriate number of classes to be used in the analysis, we applied the Akaike Information Criterion (AIC), the Bayesian Information Criterion (BIC) and the Consistent AIC (CAIC) statistics.

When interpreting the results and to avoid the potential confound with scale, we followed Lusk and Briggeman, 2009, Thomson et al., 2010, and calculated a share of preference for each milk allocation option, which is the forecasted probability that each milk allocation option *i* is picked as most important/likely. The share of preferences for the milk allocation option *i*, *S_i_*, is then defined as:

(1) $$\mathrm{ShareofPreferenceforoptioni}=S_{i}=\frac{e^{\hat{\beta_{i}}}}{\sum_{m=1}^{J}e^{{\hat{\beta }}_{m}}}$$

$\hat{\beta_{i}}$ is the forecasted probability that milk allocation *i* is picked as most important.

The shares of preferences sum to one (could also be reported on percentage basis and sum to 100%) across all nine milk allocation options. The shares of preference show the importance of one milk allocation alternative over the other alternatives on a ratio scale. This simply means that, if one milk allocation option *i* has a share of preference twice that of another milk allocation option *j*, it can be said that the milk allocation option *i* is twice as important as the milk allocation option *j* (Lusk & Briggeman, 2009).

## 3 Results

### 3.1 Socioeconomic and demographic characteristics of the sample

Table 2 summarizes the socio-economic characteristics of the sampled households. According to the results from the survey, 83% of the households were headed by men. Ninety eight percent (98%) of non-single headed households were headed by men. Sixty one percent (61%) of the households had 3–4 members, 35% had five or more members, and only 4% had two members (one of which was a child given our inclusion criteria). The average household size for the entire sample was four. The majority of household heads (80%) had a secondary school or lower educational level.

**Table 2:** Socio-economic and demographic characteristics of the sample (n = 200).

Variable	Categories	%
Sex of the household head	Male	83.0
Sex of respondent	Female	98.0
Age of the household head	18–29 years	36.0
	30–39 years	44.5
	40–49 years	13.0
	50–59 years	3.5
	60–69 years	1.5
	Above 70 years	0.5
	Not indicated	1.0
Highest education level of the household head	Primary school (grade 1–8)	30.0
	Vocational school	3.0
	Secondary school (form 1–4)	47.0
	Technical college/Diploma	18.0
	University/Degree	2.0
Marital status of the household head	Married living with spouse	84.0
	Married living separately	2.5
	Single/divorced	11.5
	Widow/widower	2.0
Primary activity of the household head	Unemployed/Retired	3.5
	Employed/laborer	69.0
	Self-employed	27.5
Number of household Members	Two	4.0
	Three	30.0
	Four	31.0
	Five	18.0
	More than five	17.0
Number of children 6–48 months living in the household	One	85.5
	Two	12.5
	Three	2.0
Household monthly income (KES)	<3,000	0.5
	Between 3,000 and 6,000	4.0
	Between 6,001 and 10,000	14.5
	Between 10,001 and 15,000	18.5
	Between 15,001 and 20,000	18.5
	Between 20,001 and 25,000	17.0
	Between 25,001 and 30,000	27.0

Given our sampling criteria, no household without a child between the age of 6–48 months or earning a household monthly income above KES30,000 was interviewed in this study.

### 3.2 Relative importance and ranking of the milk allocation options

The best-worst/most-least scores and the standardized ratio scale of the alternatives are presented in Table 3. The relative importance of each allocation alternative is also presented and the heterogeneity of responses for each alternative is evaluated by the standard deviation.

**Table 3:** Milk allocation options scores and their relative importance.

Options	Best	Worst	Best-worst scores	Std*	Sqrt (B/W)	Standardized ratio scale	Relative importance**	Ranking
O1	45	205	−0.20	0.3070	0.47	7.60	2.5%	6
O2	494	13	0.60	0.2870	6.16	100.00	33.4%	1
O3	24	235	−0.26	0.2713	0.32	5.18	1.7%	7
O4	518	17	0.63	0.3666	5.52	89.55	29.9%	2
O5	305	45	0.33	0.3850	2.60	42.23	14.1%	3
O6	12	319	−0.38	0.2651	0.19	3.15	1.0%	8
O7	239	48	0.24	0.3556	2.23	36.20	12.1%	4
O8	146	217	−0.09	0.5074	0.82	13.31	4.4%	5
O9	17	699	−0.85	0.3392	0.16	2.53	0.9%	9
Weighting factor for standardized ratio scale 16.22								
Weighting factor for relative importance 5.41								

O1: Decrease raw milk quantities for all family members without replacing it by any other food product

O2: Decrease raw milk quantities for all family members and replace it with another food product only for children < 4 years

O3: Decrease raw milk quantities for all family members and replace it with another food product for all family members EXCEPT for children < 4 years

O4: Decrease raw milk quantities for all family members and replacing it with another food product for all family members

O5: Keep raw milk quantities the same for children < 4 years and decrease it for the rest of family members

O6: Decrease the quantities of raw milk for children < 4 years without replacing it by other food products, and keep the same quantities of raw milk for adults

O7: Decrease the quantities of raw milk for children < 4 years, while replacing it by other food products, and keep the same quantities of raw milk for adults

O8: Keep buying the same quantities of raw milk by increasing milk budget

O9: Stop buying raw milk

Sqrt (B/W): Square root of the ratio of best and worst frequencies

Std*: Standard deviation

* Standard deviation from the individual scores

** Calculated from the square root of the ratio of the attribute best frequency by the attribute worst frequency and taking the highest attribute (O2) as the reference level (100%) (Loose & Lockshin, 2013)

The most preferred option of household milk allocation if prices increase by 40% was decreasing the amount of raw milk taken by all family members and substituting it with another food item only for children below the age of four years (O2). The second most preferred option was decreasing the amount of milk allocated to all family members and replacing it with another food item for all family members (O4). The least preferred option was stopping the purchase of raw milk (O9), which indicates the significance of milk in the diet of low-income households in Kenya. The ranking of options O3 and O6 among the least important ones (7th and 8th respectively) shows that the respondents are aware of the importance of milk for the growth of infants and if milk is missing or unaffordable, it should be replaced by another food product. It also shows, in combination with the previous results, that priority for milk allocation and consumption is given to infants and not to adults (for instance option O5, keep raw milk quantities the same for children below four and decrease it for the rest of family members, is ranked as third preferred option).

### 3.3 The mixed logit model estimates

Table 4 below shows the results from the mixed logit model. To avoid multicollinearity of variables, one explanatory allocation attribute had to be omitted in the maximum likelihood estimation. Option O9 (stopping purchase of raw milk) was chosen as the reference level and omitted since it was the less desired option from the best-worst scores and related importance (Table 3). As expected, all coefficients have a positive and statistically significant sign which indicates that the eight options are preferred to the base option of stopping the purchase of raw milk. The standard deviation estimates are also highly statistically significant indicating heterogeneity in preferences for milk allocation options among respondents.

**Table 4:** Maximum likelihood estimations from mixed logit model and share of preferences.

Milk options	Estimates		Share of Preferences
	Mean	Standard Deviation	SP
**O1**	2.877^***^	0.696^***^	0.016
	(0.150)	(0.132)	[0.012]
**O2**	5.594^***^	1.113^***^	0.261
	(0.200)	(0.149)	[0.177]
**O3**	2.607^***^	0.472^***^	0.012
	(0.144)	(0.169)	[0.008]
**O4**	5.843^***^	1.725^***^	0.397
	(0.218)	(0.166)	[0.257]
**O5**	4.688^***^	1.418^***^	0.137
	(0.195)	(0.143)	[0.145]
**O6**	2.315^***^	−0.566^***^	0.009
	(0.140)	(0.155)	[0.007]
**O7**	4.315^***^	1.079^***^	0.093
	(0.173)	(0.133)	[0.088]
**O8**	2.950^***^	2.409^***^	0.073
	(0.205)	(0.180)	[0.141]
**O9**	---	---	0.001
			[0.000]
Loglikelihood = 2675 Number of observations = 1800			

**, *** Statistically significant at 5% and 1% levels, respectively

Numbers in parentheses are standard errors; numbers in brackets are standard deviations

O1: Decrease raw milk quantities for all family members without replacing it by any other food product

O2: Decrease raw milk quantities for all family members and replace it with another food product only for children < 4 years

O3: Decrease raw milk quantities for all family members and replace it with another food product for all family members EXCEPT for children < 4 years

O4: Decrease raw milk quantities for all family members and replacing it with another food product for all family members

O5: Keep raw milk quantities the same for children < 4 years and decrease it for the rest of family members

O6: Decrease the quantities of raw milk for children < 4 years without replacing it by other food products, and keep the same quantities of raw milk for adults

O7: Decrease the quantities of raw milk for children < 4 years, while replacing it by other food products, and keep the same quantities of raw milk for adults

O8: Keep buying the same quantities of raw milk by increasing milk budget

O9: Stop buying raw milk (base/reference level)

The share of preference (SP) values calculated using equation (1), indicate that 39.7% of respondents would decrease raw milk quantities for all family members with replacement by another food item (probably of lower price). Option 2, corresponding to decreasing raw milk quantities for all family members with replacement with another food product only for children < 4 years was ranked second and selected by 26.1% of respondents. Option 5, which corresponds to keeping raw milk quantities the same for children < 4 years and decreasing it for the rest of family members was ranked third and selected by 13.7% of consumers. Option 4 is thrice as important as option 5. The options 9, 6, 3, and 1 have<2% of respondents who would pick any of them. These options are the most negatively impactful in terms of infant nutrition since they support a decrease in child milk consumption without any replacement by another food product.

The mixed logit share of preference estimates confirm the ranking of the best-worst scores (Table 3). Except for alternatives 4 and 2, which respectively occupy the first and second ranks compared to the second and first ranks for the best-worst scores, the other alternatives have the same ranking. However, there are differences between the importance (weights assessed by the share of preference) of each attribute which is explained by the heterogeneity in preferences between respondents.

### 3.4 Consumer segmentation

To further understand the heterogeneity indicated by the results of the standard deviations of the mixed logit estimates (Table 4), we analyzed the data using the latent class (LC) model. The Akaike Information Criterion (AIC), Bayesian Information Criterion (BIC) and Consistent Akaike Information Criterion (CAIC) results are shown in Table 5. The log likelihood decreases (improves) as the number of classes is added. AIC, CAIC and BIC values also decrease as more classes are added. However, these parameters level off after the third class (the third row in Table 5 marked in bold).

**Table 5:** Criteria for determining the optimal number of latent classes.

Classes	Log Likelihood	AIC	ΔAIC	CAIC	ΔCAIC	BIC	ΔBIC
2	−2801.49	5636.98		5710.05		5693.05	
**3**	**−2664.97**	**5381.94**	**4.52%**	**5493.70**	**3.79%**	**5467.7**	**3.96%**
4	−2586.98	5243.95	2.56%	5394.39	1.81%	5359.39	1.98%
5	−2562.83	5213.66	0.58%	5402.79	−0.16%	5358.79	0.01%
6	−2529.5	5164.99	0.93%	5392.80	0.18%	5339.8	0.35%
7	−2506.58	5137.15	0.54%	5403.65	−0.20%	5341.65	−0.03%
8	−2486.44	5114.88	0.43%	5420.06	−0.30%	5349.06	−0.14%
9	−2474.2	5108.40	0.13%	5452.26	−0.59%	5372.26	−0.43%

Three (3) clusters were considered as the most optimal number or the best cluster model given the absolute change in the AIC, BIC and CAIC values. The three-class model also has the strongest loadings of observations per cluster and, hence, comparatively easier to interpret and undertake a more nuanced analysis of the segments (Swait, 1994).

Table 6 below represents the maximum likelihood estimations for the three-class model. As in the mixed logit model, the 9th milk allocation option of stopping the purchase of milk was chosen as the reference level. The results indicate that the majority of the variables/milk allocation options present statistically significant coefficients across the three latent classes. In the third latent class, the coefficients of the attributes O2, O4, O5, O7 and O8 are statistically significant compared to the reference (O9), while those for O1, O3 and O6 are not.

**Table 6:** Latent class model estimates and shares of preference (SP) of the milk allocation options.

Options	Class 1 (65%)		Class 2 (21%)		Class 3 (14%)	
	Coefficient	SP	Coefficient	SP	Coefficient	SP
O1	4.098^***^	0.022	3.264^***^	0.024	0.354	0.090
	(0.316)		(0.419)		(0.238)	
O2	6.533^***^	0.257	5.363^***^	0.193	1.487^***^	0.280
	(0.343)		(0.441)		(0.253)	
O3	3.876^***^	0.018	3.252^***^	0.023	−0.164	0.054
	(0.316)		(0.414)		(0.263)	
O4	7.129^***^	0.466	5.006^***^	0.135	1.151^***^	0.200
	(0.356)		(0.456)		(0.254)	
O5	5.620^***^	0.103	5.154^***^	0.157	0.766^***^	0.136
	(0.343)		(0.442)		(0.240)	
O6	3.699^***^	0.015	2.789^***^	0.015	−0.341	0.045
	(0.314)		(0.415)		(0.250)	
O7	5.652^***^	0.106	4.439^***^	0.077	0.564^**^	0.111
	(0.338)		(0.434)		(0.251)	
O8	3.484^***^	0.012	6.031^***^	0.376	−1.226^***^	0.019
	(0.325)		(0.448)		(0.269)	
O9	-----	0.000	-----	0.001	-----	0.063
Log Likelihood = 2665 Number of observations = 1800						

*, **, *** Statistically significant at the 10%, 5%, and 1% levels, respectively

Numbers in parentheses are standard errors

O1: Decrease raw milk quantities for all family members without replacing it by any other food product

O2: Decrease raw milk quantities for all family members and replace it with another food product only for children < 4 years

O3: Decrease raw milk quantities for all family members and replace it with another food product for all family members EXCEPT for children < 4 years

O4: Decrease raw milk quantities for all family members and replacing it with another food product for all family members

O5: Keep raw milk quantities the same for children < 4 years and decrease it for the rest of family members

O6: Decrease the quantities of raw milk for children < 4 years without replacing it by other food products, and keep the same quantities of raw milk for adults

O7: Decrease the quantities of raw milk for children < 4 years, while replacing it by other food products, and keep the same quantities of raw milk for adults

O8: Keep buying the same quantities of raw milk by increasing milk budget

O9: Stop buying raw milk (base/reference level)

From the above latent classes, in Class 1 (65% of respondents), the results indicate that option O4 corresponding to decrease raw milk quantities for all family members with replacement by another food product for all members is the most likely to be chosen by around 47% of respondents. On average, a quarter of the respondents (25.7%) would decrease milk quantities for all family members with replacement by another food product only for children below four years (option 2). Options 7 and 5 have relatively the same importance (around 10%) but are less important compared to option 4 which is 4.4 times as important/likely to be chosen as these options. Options O4, O2 and O7 have the highest ranks, indicating that they are the most preferred/probable choices of the respondents (they aggregate around 83% of respondents’ choice). A common characteristic across the three options is that the quantities of milk allocated to children decreases when the price of milk increases and is replaced with another food item.

In Class 2 (21% of respondents) options O8, O2, O5 and O4 have the highest share of preference values and are the most likely to be chosen. Respondents in this group mainly try to keep the same quantity of milk allocated to infants below four years (O8 and O5) or try to replace it by another food product (O2 and O4) in order to keep a nutritious diet for kids. Keep buying the same quantities of milk by increasing milk budget (O8) is however the dominant option (38% of respondents will choose it) and is almost three times as important/likely to be chosen as option O4.

For respondents in Class 3 (14%), some of the options (O1, O3 and O6) present coefficients which are not statistically different from the reference level (O9). For this group, the most preferred/probable options are O2 (28% of the share of preference), O4 (20%) and O5 (14%). The former two alternatives have the consequences of decreasing the quantities of milk allocated to infants but with replacement by another food product. For the third group of respondents, it is interesting to notice that: i. buying the same quantities of milk is the least preferred/likely choice; ii. decreasing the amount of milk allocated to infants without replacement by another food item (O1, O3 and O6) is not “statistically” different from stop buying raw milk. We can infer that for this group, the quantities of milk allocated to infants are already very low and any reduction (without replacement) could seriously affect their daily required intake and growth.

For an in-depth understanding and characterization of the latent groups in Table 6, we looked at the composition of the three classes using various household sociodemographic parameters such as household size, gender of the household head, household income, education level and age of the household head (Table 7).

**Table 7:** Composition of latent classes per household characteristics.

Variable	Level	Class 1	Class 2	Class 3
Household Income* (%)	Below 10,000 KES	16.9	19.0	28.6
	10001–20000 KES	39.2	26.2	42.9
	20001–30000 KES	43.9	54.8	28.5
Gender of HH Head (%)	Male	76.9	85.7	89.3
	Female	23.1	14.3	10.7
Age of HH head^***^ (%)	18–29 years	37.7	38.1	25.0
	30–39 years	43.8	40.5	53.6
	40–49 years	13.1	16.7	10.7
	≥ 50 years	5.4	4.7	10.7
Highest Education level	Primary/Vocational school	29.5	42.5	28.0
of HH Head* (%)	Secondary school	44.9	47.5	60.0
	Technical/University	25.6	10.0	12.0
Purchasing milk (%)	Both processed & raw	60	57	43
	Raw milk only	40	43	57
Mean Raw Milk Expenditure (KES/week/HH)		313.84^a^	235.73^b^	205.18^b^
Mean Quantity of raw milk purchased (liter/week/HH)		4.00^a^	3.46^a^	2.70^b^
Mean milk consumption for children (ml/week/HH)		705.92^a^	593.13a,^b^	480.55^b^
Mean milk consumption for adults (ml/week/HH)		1012.48^a^	746.21a,^b^	636.26^b^
Number of children (6–48 months old)		1.19^a^	1.12 a	1.04 a
Mean number of children (Below 18 yrs)		2.23^a^	2.16^a^	2.10^a^
Number of adults in the household		3.17^a^	3.21^a^	3.14^a^
Household size (mean)		4.36^a^	4.33^a^	4.17^a^

*, **, *** Statistically significant at the 10%, 5%, and 1% levels, respectively

a, b Values with different superscripts are statistically significant at 10% level or less.

There are socioeconomic differences across the latent classes. Looking at household income, for instance, the plurality of households in Class 1 and the majority of households in Class 2 belong to the highest income tier (KES20,001–30,000), whereas the plurality of households in Class 3 belong to the middle-income tier (KES10,001–20,000).

Households in Class 2 chose increasing milk budgets in order to maintain the quantities of milk they purchase as their best option. Their second best option is keeping the same milk quantities given to children and decreasing milk allocation for the rest of the family. Looking at the household characteristics above, we find that 55% of the households in this class are on the highest income tier compared to the other two classes. For those in Class 3, the plurality of the households are in the middle-income tier. Comparatively, it also has a bigger proportion of household in the lowest incomer tier (29%). Their most preferred option is decreasing the amount of milk allocated to all family members but replacing with another food item for children only. This indicates that households with higher incomes can afford to either purchase substitutes or increase budgets in order to keep the quantities of milk required by their households. Milk allocation to children would, therefore, be mostly affected for poorer households.

### 3.5 Comparison with households’ current decision

This study was conducted during the dry season (April–May) when milk availability is normally lower than usual and milk prices are higher than the rest of the year. Using similar allocation options like the ones used in the best-worst experiment, we asked respondents what decisions they have taken in terms of milk purchase and household milk allocation. When milk was reported to have been replaced by another food item in the diet, we asked the respondents to specify the substitute product. Table 8 below summarizes the responses.

**Table 8:** Proportion of households having adopted specific milk allocation decision.

No.	Options	%
D1	Decreased raw milk quantities for all family members without replacing it by any other food product	18
D2	Decreased raw milk quantities for all family members and replaced it with another food product only for children < 4 years (specify the product)	8.5
D3	Decreased raw milk quantities for all family members and replaced it with another food product for all family members EXCEPT for children < 4 years	0
D4	Decreased raw milk quantities for all family members and replaced it with another food product for all family members (specify the product)	13
D5	Kept raw milk quantities the same for children < 4 years and decreased it for the rest of family members	4
D6	Decreased the quantities of raw milk for children < 4 years without replacing it by other food products, and kept the same quantities of raw milk for adults	2
D7	Decreased the quantities of raw milk for children < 4 years, while replacing it by other food products, and kept the same quantities of raw milk for adults	0
D8	Kept buying the same quantities of raw milk by increasing milk budget	48.5
D9	Stopped buying raw milk and replaced by other food product(s) (specify the product)	1.5
D10	Stopped buying raw milk without replacing it by another food product	0

Although almost half (48.5%) of the households were maintaining purchase of milk by increasing milk budget allocation after a price increase of around 20%, increasing the milk price by 100% from rainy season prices (≈ KES 50/liter) to the best-worst scenario prices (KES 100/liter), would result to only 7.3% (Table 4) maintaining this choice. The majority would tend to shift to alternatives that reduce the amount of milk bought and consumed by the household members. This is not surprising given that 47% of the households were already decreasing milk consumption given the high milk prices (Table 8). Infants were also affected by the increase in milk prices since 41% (D1, D2, D4, D6 and D9) of the households indicated that they have decreased the amount of milk allocated to infants, and in 20% of the cases it was without any replacement by another food item. The households that were substituting the decreased amount with other food were often compensating the amount with fruits and/or porridge, either for children or for the entire household (Table 9).

**Table 9:** Households milk substitution.

Substitute	D2		D4	
	Freq.	%	Freq.	%
Fruits/Fruit juice	5	26	9	32
Porridge	11	58	16	57
Cooked bananas or Potatoes	2	11	0	0
Black tea/ Drinking chocolate/Cocoa	0	0	3	11
Yoghurt	1	5	0	0
**Total**	**19**	**100**	**28**	**100**

D2: Decrease raw milk quantities for all family members and replace it with another food product only for children < 4 years

D4: Decrease raw milk quantities for all family members and replacing it with another food product for all family members

## 4 Discussion

In this study, we tested the relative importance of various intrahousehold milk allocation choices in a hypothetical event of milk price increase. Such increase could be occasioned by, among other things, elimination of comparatively cheaper unprocessed milk currently provided by the informal dairy sector. The findings from the best-worst choice experiment indicate that an increase in milk price will affect milk allocation to and intake by children within the household. Although stopping the purchase of raw milk was the least preferred choice for all the respondents, and therefore an unlikely event, overall intake of milk allocated to infants would decrease with an increase of milk price.

Generally, households in the lowest income tier were more likely to be affected by any upward adjustments to milk prices than the relatively wealthier ones. Schneider (2018), using data collected from the same households as in this study, found that households would increase their demand for dairy products by 9.4% if their income increased by 10% and decrease their demand for dairy products by 6.3% if prices increased by 10%. Raw milk was among the most sensitive products to changes in price. In a *meta*-analysis conducted to estimate effect of price increase on food consumption, it was established that increasing the price of dairy products by 10% resulted in a reduction of consumption by 7.2% in low- and middle-income countries (Cornelsen et al., 2015). This may translate into low-income households drastically reducing consumption of milk, which has important implications for nutrient intake given the beneficial nutrient profile milk has. Milk contains proteins of very high biological value (i.e. has a good profile of essential amino acids that can be readily incorporated into the body’s protein) and is a good source of vitamins A, B1, B2, B12 and D, as well as minerals like calcium, phosphorus, and zinc, involved in bone health and growth. Therefore, opting for cheaper food items that might not be of equivalent nutritional value to milk for a sustained length of time can have profound consequences in the nutritional status of children. A Randomized, controlled food-feeding study in Kenya showed that supplementation with milk in younger and stunted children has a greater rate of gain in height (Neumann et al., 2007).

The results of this study indicate that the overall milk allocated to infants will decrease in most of the cases with replacement by another food item (options O4, O2, O7). Households try to substitute the lack of nutrient value from decreasing milk consumption by other affordable products and tend to prioritize children when it comes to distributing the nutritional value of food (i.e. the keep the amount of milk, or substitute by other food item only for children). It is worth noting that milk is associated with improved growth and cognitive development and is, therefore, an important diet component in areas with reported high child stunting rates as the one in our study (de Beer, 2012, Neumann et al., 2007) where important pockets of undernutrition have been found (Dominguez-Salas et al., 2016). Lower household milk consumption and allocation to children is likely driven by budget constraints. Notably, the option of stopping to buy milk has always been ranked the lowest/least probable which confirms the importance of milk consumption in Kenyans’ diet.

Options O5 (keep raw milk quantities for children < 4 years and decrease it for the rest of the family members) and O8 (keep buying the same quantities of milk by increasing milk budget), both together sum only to 21% (mixed logit estimates) of best/most probable options selected by respondents. At the group levels (latent class estimates), the shares of preferences for options O5 and O8 together are even lower for Class 1 (11.5%) and Class 3 (15.5%) respondents (who represent 79% of total respondents). For Class 2 respondents, maintaining the same quantities of milk allocated to infants is more likely with an aggregate share of preference of (53.3%) mainly spurred by option O8 on keeping the same quantities by increasing milk budget (37.6%). Class 2 has the highest percentage (54.8%) of respondents in the upper income bracket (KES 20,001–30,000).

Considering our findings and given that milk intake in Kenya is low compared to the recommended amount, there is a need to include strategies for promoting and facilitating access to milk for low-income consumers in any policy intervention aiming at developing the sector, ensuring greater compliance with food safety standards and shifting informal market share to the formal value chain. As Argwings-Kodhek et al. (2005) argued, in order to reach global requirements, policy initiatives geared towards improving milk consumption should design pricing mechanisms that enhance availability and access of dairy products.

The results from the decisions taken by households during the survey period (Table 8) when milk prices were already high because of the dry season showed similarities and coherence (overall tendency of decreasing household milk purchase and allocation to infants) with their responses to the best-worst experiment.

When asked about the type of food item households are currently using as a substitute for the decrease in milk consumption, fruits and porridge were the most cited ones. Fruits are rich in vitamins and minerals, as well as fiber. But milk covers a broad range of nutrients in a single commodity, while different fruits can have complementary nutrient profiles. Vitamin B12 is essential in animal source foods and there would not be a replacement for it in fruits. Also, nutrients in milk tend to be more bioavailable, like calcium or vitamin A. Porridge, unless it is made of fortified flour (not always the case in Kenya despite the legal requirement), is a good source of carbohydrates, but less so of micronutrients, particularly if they are refined (i.e. important loss of micronutrients).

## 5 Conclusions and policy implications

The results of the study indicate that any policy change that may result in an increase in milk prices would decrease overall milk demand and consumption in low-income households. In addition, household decisions are directly affected (reduce) when it comes to the amount of milk allocated to children below the age of four years. Although they may compensate for the decreased amount allocated to children by substituting with other food items, the nutritional value of the replacement, taste and preference, complementarity and “nutrient price” among other factors is unlikely to match that of milk.

Any reforms to policies and regulatory systems aimed at streamlining the dairy sector should account for responsiveness of consumers to price variations, preferences and intrahousehold allocations, in order to avoid potential harm from adoption of poorer diets. In light of our findings and households response to increases in milk prices, we recommend that regulatory and development agencies should consider policies that will not result in great increases in the market price of milk (or other nutritious products), and that such policies should be only implemented alongside activities to promote consumption of affordable, accessible and safe milk for children and the entire household. Moreover, policies promoted on the grounds of public health should be evidence- and risk-based, using ex-ante assessments to measure the health burden associated with milk-borne illnesses, which is primarily driven by food consumption habits. In urban and *peri*-urban areas in Kenya, and in many other East African countries where milk is widely boiled before consumption and high-quality protein product largely unaffordable, we argue that the costs to nutritional security derived from restrictions and bans to informal milk markets and the consequent increase in market milk price would outweigh the marginal reduction in the public health impact derived from consumption of milk from informal markets.

Secondly, given our study findings that overall household demand for milk decreased in a period of high milk prices compared to when prices were lower, it is important for dairy policies to consider milk affordability within the context of economic growth in order to safeguard nutrition security of children. This may involve policies and interventions targeting smallholder milk producers that improve productivity (access to new technologies, improved breeds, feeds and forages, etc.), and interventions targeting both smallholder milk producers and small-scale milk vendors that improve milk safety and minimize spoilage and losses along the supply chain (cooling machines, facilitated access to adequate milk containers like the Mazzican, trainings on milk handling and storage, etc.).

Thirdly, there is a need to strengthen household resilience to milk price variations. Considering that a bigger proportion of the respondents would replace unaffordable milk with other food items, often porridge and fruits, we recommend the identification and creation of public awareness on food substitutes that offer similar or better nutritional value (nutrient-dense protein rich) at similar or lower purchase and preparation costs. This cushions the welfare of the child not only in the event of price variation, but also in times of scarcity that may be attributed to distal factors beyond policy change.

Further, we reiterate that in order to assess the likely effect of milk price variation on household allocation to children, it is important to factor the heterogeneity at the households. As an area that this study explored, we found that characterizing the differences between various groups as per their milk allocation choices is important as it highlights the implications on children’s milk intake. We demonstrated that looking at the differences of households in terms of their demographic and socioeconomic characteristics offers more nuanced information that can be useful in designing more responsive policies.

Many countries in sub-Saharan Africa look at Kenya as a progressive country on matters of dairy and benchmark its policy and dairy development pathway. Some of those countries like Rwanda, Tanzania, and Malawi, with comparatively lower per capita consumptions, have attempted to eliminate informal markets from their dairy sectors. This could potentially pose similar issues demonstrated by this study (Revoredo-Giha and Toma, 2016, Wegerif and Martucci, 2019). As they adopt and contextualize dairy policies, we recommend they also consider estimating and addressing the likely effects of such policies in terms of diets; otherwise well-intended policies will likely milk the dairy sectors and households dry.

Finally, given that this study is an ex-ante assessment using experimental economics premised on a hypothetical scenario, the projected results are illustrative and only show the possible effects of a policy change eliminating cheaper milk trade on children’s nutrition as far as milk intake is concerned. It does not encompass the likely effect of such policy change on the supply end in dairy markets. As such, we recommend that research should explore, using for instance ex-ante impact assessment, the likely effects of such policy change in decreasing producer and trader revenues as a result of decreased intake at the household level. This is much needed evidence by policy makers in Africa, and low- and middle-income countries in general, to understand more holistically the effects that modernizing value chains and enforcing compliance with international standards may have on product price and the related consumer response, as well as the effect such policies will have on the business revenues and livelihoods of various actors along the value chain.

Ethical Considerations

Ethical approval for the research methodology and the tools was obtained from the International Livestock Research Institute – Institutional Ethics Research Committee (ILRI-IREC) (ILRI-IREC2018-17. ILRI-IREC is accredited by the National Commission for Science, Technology and Innovation in Kenya. Permission to conduct the survey was also obtained from NACOSTI and local authorities. Prior to each interview, the study’s objectives and respondents’ rights were explained before seeking their consent to participate. Verbal and written consent to participate in the study were obtained before initiating discussions and a signed copy of the consent form retained by both parties.

Funding sources

This publication is based on research funded by the Bill & Melinda Gates Foundation and UK Aid from the UK government (Ref. OPP1156625). The findings and conclusions contained within are those of the authors and do not necessarily reflect positions or policies of the Bill & Melinda Gates Foundation or the UK government. The work also received financial support from the CGIAR Research Program on Agriculture for Nutrition and Health.

## CRediT authorship contribution statement

**Emmanuel Muunda:** Methodology, Software, Formal analysis, Investigation, Writing - original draft. **Nadhem Mtimet:** Conceptualization, Methodology, Software, Validation, Formal analysis, Investigation, Writing - original draft, Visualization, Supervision, Funding acquisition. **Franziska Schneider:** Data curation, Investigation. **Wanyoike Francis:** Software, Data curation. **Paula Dominguez-Salas:** Resources, Writing - review & editing. **Silvia Alonso:** Conceptualization, Resources, Writing - review & editing, Supervision, Project administration, Funding acquisition.

## Declaration of Competing Interest

The authors declare that they have no known competing financial interests or personal relationships that could have appeared to influence the work reported in this paper.

## Appendix A . Most-Least score calculation

(a) $$Standardized\left(Most-Least\right)Score=\frac{N_{\mathrm{Most}}-N_{\mathrm{Least}}}{m\times n}$$

$N_{\mathrm{Most}}$: number of times the allocation alternative was chosen as most likely

$N_{\mathrm{Least}}$: number of times the allocation alternative was chosen as least important

m: number of respondents = 200

n: number of times the allocation alternative was presented to each respondent = 4

Positive values for the difference between Most and Least mean that the given attribute was chosen more frequently as ‘‘Most’’ than ‘‘Least’’ and negative scores mean the opposite. The negative scores do not show negative importance but indicate a below average preferences.

In order to allow for a more intuitive interpretation, we transformed the scores to a positive scale by using the square root of the best divided by the worst as suggested by Lee et al. (2008). We then standardized the positive scales where the most important milk allocation alternative took the value of 100 by multiplying each positive scale using the weighted factor of 16.22 obtained using the formula below (Loose & Lockshin, 2013):

(b) $$\mathrm{Weighting}\;\mathrm{factor}\;\mathrm{for}\;\mathrm{standardized}\;\mathrm{ratio}\;\mathrm{scale}=\frac{100}{\max\left(sqrt\frac{B}{W}\right)}$$

This way, we interpreted all the least preferred milk allocation choices as a ratio relative to the most preferred alternative. For confirmation and further simplicity of interpretation, we standardized the square-root scale to add up to 100% using a factor of 5.41 calculated using the formula below:

(c) $$\mathrm{Weighting}\;\mathrm{factor}\;\mathrm{for}\;\mathrm{relative}\;\mathrm{importance}=\frac{100}{\sum_{n=1}^{i}\left(\mathrm{sqrt}\frac{B}{W}\right)}$$

## Appendix B . Models estimations

### Maxdiff model

Maxdiff model derives from the Random Utility Theory (Thurstone, 1927) and the theory of Lancaster (1966). The n^th^ respondent indirect utility, *U*, derived from the selected best-worst pair in each choice card *t*, is the difference in utility between the *i* best and the *j* worst options plus the error term (Caputo & Lusk, 2020):

$U_{\mathrm{nit}}=\beta_{\mathrm{it}}-\beta_{\mathrm{jt}}+{∊}_{\mathrm{nit}}$ (d)

Where β is the vector of estimated importance parameters of the best and worst options (*i* and *j* respectively) relative to a base option normalized to zero to ensure estimation of the model (option 9 in this study).

The probability that allocation alternative *i* is preferred over alternative *j* for a choice card *t* is formulated as (Loose & Lockshin, 2013):

$P\left(i/i,j\right)=P\left(v_{\mathrm{it}}+\varepsilon_{\mathrm{it}}\right)>P\left(v_{\mathrm{jt}}+\varepsilon_{\mathrm{jt}}\right)$ (e)

In the above formula, *v* represents the observable utility component (the milk allocation alternative) and ε represents the stochastic error term. Different econometric models could be estimated depending on the assumption of the error term. If the error term is independently and identically distributed (iid), then we have the multinomial logit model (Lusk & Briggeman, 2009):

$Pr\left(iwaschosenbestandjwaschosenworst\right)=P\left(i,j\right)=\frac{e^{\left(\beta_{\mathrm{nit}}-\beta_{\mathrm{njt}}\right)}}{\sum_{l=1}^{J}\sum_{m=1}^{J}e^{(\beta_{\mathrm{nlt}}-\beta_{\mathrm{nmt})}}-J}$(f)

### Mixed logit model

In this study we estimated the mixed logit model and the latent class model. For the mixed logit model, we assume that β is distributed normally with mean *b* and covariance *w*. The unconditional probability that an individual n selects option *i* as best and option *j* as worst is expressed as:

$P_{n(i,j)}=\int_{\beta }^{0}\prod_{t=1}^{T}\frac{e^{\left(\beta_{\mathrm{nit}}-\beta_{\mathrm{njt}}\right)}}{\sum_{l=1}^{J}\sum_{m=1}^{J}e^{(\beta_{\mathrm{nlt}}-\beta_{\mathrm{nmt})}}-J}f\left(\beta_{n}\backslash b,w\right)d\beta_{n}$ (g)

where $f(\beta_{n})$ is the density of the importance parameters $\beta_{n}$related to the different options of milk allocation presented to the respondents. The 9th parameter vector was normalized to 0 to ensure identification of the model (Ouma et al., 2007). The model was estimated using simulations (Train, 2009) at 500 pseudo Halton draws using the software STATA (14.0).

### Latent Class model

To identify possible sources of heterogeneity, we applied the latent class (LC) model (Goodman, 1974, Lazarsfeld, 1950) that intrinsically sort individuals into a number of classes. Each of these classes is characterized by homogenous preference within class, while heterogeneity exists across classes (Boxall & Adamowicz, 2002). Within each class, however, it was assumed that choices of alternatives by individuals from one choice scenario to the other was independent and the choice probabilities were generated through a multinomial logit model. Following the LC modeling, the probability that individual n chooses option *i* as the best/most likely and option *j* as the worst/least likely for a choice card t , given that the individual belongs to the LC *c* is specified as follows:

$P_{n(i,j)/c}=\prod_{t=1}^{T}\frac{e^{\left(\beta_{\mathrm{nit}/c}-\beta_{\mathrm{njt}/c}\right)}}{\sum_{l=1}^{J}\sum_{m=1}^{J}e^{(\beta_{\mathrm{nlt}/c}-\beta_{\mathrm{nmt}/c)}}-J}$ (h)

$\beta_{\mathrm{nit}/c}$ is a class-specific parameter (for class *c* in this case) that captures heterogeneity in preference across classes. However, classes are not observable, and we therefore estimate probabilities of households belonging to respective classes using a multinomial logit model as per equation (f).
